# Growth arrest and apoptosis via caspase activation of dioscoreanone in human non-small-cell lung cancer A549 cells

**DOI:** 10.1186/1472-6882-14-413

**Published:** 2014-10-24

**Authors:** Pintusorn Hansakul, Kalaya Aree, Sermkiat Tanuchit, Arunporn Itharat

**Affiliations:** Division of Biochemistry, Department of Preclinical Science, Faculty of Medicine, Thammasat University, Rangsit Campus, Khlong Luang, Pathum Thani, 12120 Thailand; Division of Microbiology, Department of Preclinical Science, Faculty of Medicine, Thammasat University, Rangsit Campus, Khlong Luang, Pathum Thani, 12120 Thailand; Research Administration Office, Faculty of Medicine, Thammasat University, Rangsit Campus, Khlong Luang, Pathum Thani, 12120 Thailand; Department of Applied Thai Traditional Medicine Center, Faculty of Medicine, Thammasat University, Rangsit Campus, Khlong Luang, Pathum Thani, 12120 Thailand

**Keywords:** Dioscoreanone, *Dioscorea membranacea*, Antiproliferative, Apoptosis, Caspase activation

## Abstract

**Background:**

Dioscoreanone (DN) isolated from *Dioscorea membranacea* Pierre has been reported to exert potent cytotoxic effects against particular types of cancer. The present study was carried out to investigate the cytotoxicity of DN against a panel of different human lung cancer cell lines. The study further examined the underlying mechanisms of its anticancer activity in the human lung adenocarcinoma cell line A549.

**Methods:**

Antiproliferative effects of DN were determined by SRB and CFSE assays. The effect of DN on cell cycle distribution was assessed by flow cytometric analysis. Apoptotic effects of DN were determined by sub-G_1_ quantitation and Annexin V-FITC/PI flow cytometric analyses, as well as by changes in caspase-3 activity and relative levels of Bax and Bcl-2 mRNA.

**Results:**

DN exerted antiproliferative and cytotoxic effects on all three subtypes of non-small cell lung cancer (NSCLC) cells, but not on small cell lung cancer (SCLC) cells and normal lung fibroblasts. DN slowed down the cell division and arrested the cell cycle at the G2/M phase in treated A549 cells, leading to a dose- and time- dependent increase of the sub-G1 population (apoptotic cells). Consistently, early apoptotic cells (AnnexinV ^+^/PI^-^) were detected in those cells that were treated for 24 h and increased progressively over time. Moreover, the highest activity of caspase-3 in DN-treated A549 cells was detected within the first 24 h, and pretreatment with the general caspase inhibitor z-VAD-fmk completely abolished such activity and also DN-induced apoptosis in a dose-dependent manner. Additionally, DN increased the Bax/Bcl-2 ratio in treated A549 cells with time, indicating its induction of apoptosis via the mitochondrial pathway.

**Conclusions:**

This study reveals for the first time that the anticancer activity of DN was induced through regulation of the Bcl-2 family protein-mediated mitochondrial pathway and the subsequent caspase-3 activation in A549 cancer cells, thus supporting its potential role as a natural apoptosis-inducing agent for NSCLC.

## Background

Lung cancer is a leading cause of cancer-related deaths worldwide [1], and it can be divided into two histological groups: non-small cell lung cancer (NSCLC) and small cell lung cancer (SCLC). NSCLC accounts for almost 80% of lung cancer cases and consists mainly of adenocarcinoma, squamous cell and large cell carcinoma. Although many factors, e.g., tobacco smoke, ionizing radiation and viral infection are known to increase the risk of this cancer type, the mechanisms involved in lung cancer formation remain largely unknown to date [2]. Despite improvements in tumor response to chemotherapy, the overall survival rate for lung cancer patients in advanced or late stages of the disease is still low and has not improved significantly in recent past decades [1]. Because of the mutation heterogeneity of lung cancer cells associated with acquired drug resistance, the search for novel anticancer agents with an enhanced specificity for cancer cells remains an urgent need in order to increase the potency of chemotherapy [3]. Herbal medicines in the treatment of cancer as an alternative therapy have long been an important source for such agents, some of which are currently used in clinical practice [4]. In recent years, plant-derived compounds have been extensively screened to explore their potential for the development of new anticancer drugs.

Exploring the precise molecular mechanisms involved in actions of anticancer agents has become an important approach for anticancer drug evaluation and development. Among these molecular mechanisms, apoptosis is a highly regulated process of cell death that serves to eliminate heavily damaged cells without injuring surrounding healthy cells, and its dysregulation underlies numerous pathological conditions including cancer [5]. Over the years, apoptosis has emerged as the major mechanism by which anticancer agents act to eliminate cancer cells [6]. Recent clinical data have revealed that anticancer drugs that restore deregulated apoptosis or apoptosis-related signaling pathways in cancer cells can significantly improve patient survival for patients who have contracted various advanced cancer diseases [7]. In this context, several plant compounds have been proven, in a more specific manner, to possess promising anticancer activity through their apoptosis-inducing effects [8].

**Figure 1:**
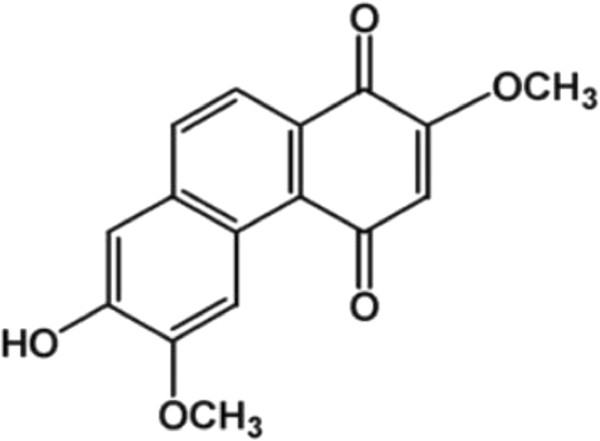
**Structure of dioscoreanone (DN).**

Dioscoreanone (DN) is 1, 4-Phenanthraquinone (Figure 1) isolated from the ethanolic extract of dried rhizomes of *Dioscorea membranacea* Pierre [9], which has long been used in various traditional Thai herbal remedies for cancer and inflammatory diseases. Previous studies have shown the selective anticancer and anti-inflammatory activities of this herbal plant [10, 11], providing the scientific support for its traditional uses. Moreover, DN was demonstrated to exert selective cytotoxic effects against human lung and breast cancer cell lines, but not against normal cells [9]. However, the molecular mechanisms underlying its cytotoxicity have not yet been explored.

In the present study, we first examined dose–response growth inhibitory and cytotoxic effects of DN on lung cancer cells including NSCLC and SCLC versus normal human lung fibroblasts. We further investigated apoptosis-involved mechanisms underlying the anticancer activity of DN in human lung adenocarcinoma A549 cells.

## Methods

### Dioscoreanone preparation

Rhizomes of *Dioscorea membranacea* Pierre ex Prain & Burkill were extracted with 95 % ethanol to obtain crude extract as previously mentioned [11]. This plant was collected from Amphoe Patue, Chumphorn Province. Authentication of plant material was carried out at the herbarium of the Department of Forestry, Bangkok, Thailand (Voucher number SKP A062041305). Dioscoreanone (DN) was isolated from the crude ethanolic extract using previously described methods [10]. The structure of DN (Figure 1) was confirmed by comparing its structure with previously reported ^1^H- and ^13^C-NMR spectral data [9]. The stock solution of DN was prepared in DMSO at a concentration of 35 mM. The final concentration of DMSO was at or below 0.1% in all experiments.

### Cell culture

Five cell lines were purchased from American Type Culture Collection (ATCC; Rockville, MD, USA), namely three subtypes of human non-small cell lung cancer (NSCLC) consisting of A549 (adenocarcinoma), COR-L23 (large cell carcinoma), and NCI-H226 (squamous cell carcinoma); human small cell lung cancer (SCLC) in the form of NCI-H1688; and human normal lung fibroblasts as MRC-5. All cancer cell lines were maintained in RPMI-1640 supplemented with 10% FBS, and MRC-5 cells were cultured in MEM supplemented with 10% FBS, 1 mM nonessential amino acid, and 1% sodium pyruvate at 37°C in 5% CO_2_ incubator.

### Cell proliferation assay

Antiproliferative effects of DN were measured by the SRB assay. Briefly, cells were seeded in a 96-well plate containing 100 μl of culture medium. Cell numbers are indicated in the bracket (A549 = 1 × 10^3^ cells/well; COR-L23 and NCI-H226 = 3 × 10^3^ cells/well; NCI-H1688 and MRC-5 = 2 × 10^4^ cells/well). On the following day, one plate of each cell line was fixed *in situ* with 100 μl of 10% (w/v) cold trichloroacetic acid (TCA), and it was stained with 0.4% (w/v) sulforhodamine B (SRB) in 1% acetic acid solution as a means of representing the cell number at the time of adding DN (zero hour). One hundred microliters of DN was added to the tested plates to obtain final concentrations of 0.35, 3.5, 17.5 and 35 μM, and these plates were incubated for additional 72 h at 37°C with 5% CO_2_. At the end of 72 h, the medium was removed, and the cells were gently fixed with 10% (w/v) cold TCA. The cell numbers were estimated indirectly by staining the total cellular protein content of each well with 0.4% (w/v) SRB. The optical density (O.D.) was measured at 492 nm. The percentage of cell survival for each concentration was calculated using the following formula:


or


where T is the average O.D. of cells treated with DN for 72 h; T_0_ is the average O.D. at 0 h; C is the average O.D of cells treated with media only for 72 h. Based on the formula, the percentage of cell survival can be greater than zero, zero or less than zero. Dose–response curves were plotted as percentage of cell survival versus DN concentration. The concentrations of DN required for 50% growth inhibition (GI_50_), total growth inhibition (TGI), and 50% loss of cells (lethal concentration, LC_50_) relative to the untreated cells were obtained by interpolating dose–response curves with cubic spline using GraphPad Prism 4.0 Software (GraphPad Software, Inc., USA).

### CFSE proliferation assay

Carboxyfluorescein diacetate, succinimidyl ester (CFDA-SE) is a cell-tracking dye used to label cells for examining their proliferative activity. It diffuses into the cytoplasm where its acetate groups are cleaved to yield a highly fluorescent derivative (CFSE) that is retained in the cell. A profile of sequential halving of CFSE fluorescence intensity with each generation can be monitored, allowing the visualization of the number of rounds of cell division. Inhibition of cell division by any substance can thus be traced through changes in CFSE profile. In the assay, detached A549 cells were labeled with 10 mM carboxyfluorescein-succinimidyl ester (CFSE) (BD bioscience, USA) at 37°C for 20 min in the dark and washed twice with PBS containing 10% FBS (FACS buffer) to remove excess CFSE. Cells were plated at 1 × 10^5^ cells/well in 24-well plates and incubated at 37°C with 5% CO_2_. At least 12 h after seeding, 30 μM dioscoreanone was added into each well, and the cells were further incubated for 24, 48 and 72 h. At the appropriate point in time, cells were detached, washed twice, re-suspended in FACS buffer, and analyzed immediately using a FACSCalibur flow cytometer (Becton Dickinson, USA). Numbers of cell division as well as two parameters, e.g. proliferation index and precursor frequency, were analyzed using ModFit LT 3.2 program (Verity Software House, USA). Proliferation index was calculated as the sum of cells in all generations divided by the number of original parent cells; this index is useful for determining antiproliferative effects of DN on a population of cells. On the other hand, precursor frequency was calculated as the percentage of cells in the original parent population that proliferated in response to DN.

### Cell cycle analysis

The percentage of cells in sub-G1 (apoptotic cells), G1, S and G2/M phases was determined by DNA flow cytometry. Briefly, A549 cells were plated at 1 × 10^5^ cells/well in 24-well plates and incubated with 15 μM and 30 μM DN for 24, 48 and 72 h at 37°C with 5% CO_2_. After treatment, cells were collected by trypsinization, fixed gently (drop by drop) in 80% ethanol, and then stored at -20°C overnight. Then, cells were washed with phosphate-buffered saline (PBS) and stained with 0.5 ml PI/RNase staining buffer (BD bioscience, USA) for 30 min at room temperature in the dark. These stained cells were collected using a FACSCalibur flow cytometer (Becton Dickinson, USA) and analyzed for cell cycle phases with ModFit LT 3.2 Software (Verity Software house, USA). Cell cycle distribution was also determined in DN-treated cells after pretreatment for 1 h with the pancaspase inhibitor z-VAD-fmk, which was added to final concentrations of 2.5 and 25 μM.

### Annexin-V/PI double staining assay

Flow cytometry was used to discriminate between intact and apoptotic cells. A549 cells were stained with fluorescein isothiocyanate (FITC) labeled annexinV that binds to membrane phosphatidylserine and with propidium iodide (PI) that binds to cellular DNA according to the manufacturer’s instructions (BD bioscience, USA). Briefly, A549 cells were plated at 1 × 10^5^ cells/well in 24-well plates. After exposure to 15 μM and 30 μM DN for 24, 48 and 72 h, cells were trypsinized, washed with cold PBS, and resuspended in 100 μl of binding buffer containing 5 μl of FITC Annexin V and 5 μl of PI. Then cells were gently vortexed and incubated for 20 min at room temperature in the dark. Four hundred microliters of binding buffer was added to each tube. Cells were then collected using a FACSCalibur flow cytometer (Becton Dickinson, USA) and analyzed with CellQuest Software (BD bioscience, USA).

### Caspase-3 activity assay

Activity of caspase-3 was detected using the CaspACE™ Assay System (Promega, USA). Briefly, A549 cells were plated at 1 × 10^5^ cells/well in 24-well plates. After exposure to 30 μM DN for 24, 48 and 72 h, control or treated cells were lysed in 50–100 μl of cold lysis buffer by sonication, and the mixture was incubated on ice for another 20 min. The cell lysates were centrifuged at 15,000 × g for 20 min at 4°C, and the supernatant fraction was collected. The assay was performed in a total volume of 100 μl in 96-well plates. Cell extracts with an equal amount of protein (50–100 μg of total protein) were added to each reaction containing caspase assay buffer and specific colorimetric substrate (DEVD-pNA) for caspase-3, and the mixture was incubated at 37°C for another 4 h according to the manufacturer’s protocol. The absorbance was measured at 405 nm. Caspase-3 activity was also measured in cell extracts treated with DN after pretreatment for 1 h with the pancaspase inhibitor z-VAD-fmk, which was added to a final concentration of 25 μM.

### Real-time Quantitative PCR Analysis

After exposure to 30 μM DN for 24, 48 and 72 h, total RNA was isolated from A549 cells using the total RNA extraction kit (Real Biotech Corporation, Taiwan). Two hundred and fifty nanograms of input total RNA was converted to single-stranded cDNA using High Capacity cDNA Reverse Transcription Kit (Applied Biosystems, USA). Bcl-2 and Bax gene expression analyses were performed using the Applied Biosystems (ABI) StepOne™ and StepOne Plus™ Real-Time PCR System (The Applied Biosystems, USA) as well as commercially available primer/probe sets, which are pre-designed FAM™ dye-labeled TaqMan® MGB (minor groove binder) probe and primer sets (inventoried Taqman® Gene Expression Assays) for human BCL2, BAX and GAPDH genes. The thermal cycling parameters were one cycle of 50°C for 2 min, one cycle of 95°C for 10 min, 40 amplification cycles of 95°C for 15 sec, and 60°C for 1 min. Relative quantification of gene expression was performed using the comparative threshold (C_T_) method according to the 2^-ΔΔCT^ method as described by the manufacturer. The C_T_ values of target genes (Bcl2 and Bax) in each sample set (ΔC_T_ test sample) were normalized to those of GAPDH. The ΔΔC_T_ is calculated by the formula ΔΔC_T_ = ΔC_T_ test sample – ΔC_T_ control. ΔΔC_T_ was converted to fold changes in mRNA gene expression relative to control by the equation 2^-ΔΔCT^. The fold changes are presented as the mean ± SD.

### Statistical analysis

All experiments were performed independently at least three times, and the results were expressed as the mean ± SD. Statistically significant differences among the groups were analyzed by one-way analysis of variance (ANOVA), followed by post-hoc analysis. *p* value <0.05 was considered statistically significant (SPSS 16.0 for Windows).

## Results

### The antiproliferative and cytotoxic effects of DN in several lung cancer cell lines

In this study, we further evaluated the antiproliferative effect of DN in two major types of human lung tumor-derived cell lines versus the human lung fibroblast cell line MRC-5. These two major types were NSCLC cell lines that included three main subtypes, namely A549 (adenocarcinoma), COR-L23 (large cell carcinoma), and NCI-H226 (squamous cell carcinoma), as well as a SCLC cell line, NCI-H1688. Our results showed that DN in the concentration range of 0.35-35 μM exerted a significant dose-dependent inhibition of cell growth in all cell lines tested. As a result, the concentrations of DN required for 50% growth inhibition (GI_50_), total growth inhibition (TGI), and 50% loss of cell numbers (lethal concentration, LC_50_) at 72 h were thus able to be assessed as shown in Table 1. Paclitaxel was also used as a positive control.

**Table 1:** **Cytotoxic effects of dioscoreanone (DN) on human lung cell lines**

Cell line	Lung cell type	Paclitaxel (μM)			DN (μM)		
		GI _50_	TGI	LC _50_	GI _50_	TGI	LC _50_
MRC-5	Normal lung fibroblast	0.016 ± 0.005	0.297 ± 0.008	>32	11.4 ± 1.3	19.6 ± 0.7	>32
	3 subtypes of NSCLC						
A549	Adenocarcinoma	0.016 ± 0.001	0.058 ± 0.012	2.800 ± 0.400^a^	6.2 ± 0.7^a^	10.3 ± 0.3^a^	15.7 ± 1.3^a^
NCI-H226	Squamous cell carcinoma	0.017 ± 0.001	4.898 ± 0.480^a^	20.672 ± 0.436^a^	5.7 ± 1.2^a^	9.8 ± 0.3^a^	13.6 ± 1.2^a^
COR-L23	Large cell carcinoma	0.017 ± 0.001	3.450 ± 0.167^a^	17.725 ± 0.084^a^	3.7 ± 0.9^a^	8.0 ± 1.4^a^	18.9 ± 2.5^a^
NCI-1688	SCLC	0.012 ± 0.001	20.560 ± 0.147^a^	>32	15.2 ± 2.1	21.1 ± 2.4	>32

DN concentrations required for 50% growth inhibition (GI_50_), total growth inhibition (TGI), and 50% decrease in cell numbers (LC_50_) relative to the untreated cells as measured by the SRB assay. Data are presented as the mean ± SD of at least 3 independent experiments.

^a^Statistical significance (*p* <0.05) versus MRC-5.

DN exhibited a significant growth-inhibitory effect in all three subtypes of NSCLC cell lines with GI_50_ values ranging from 3.7 to 6.2 μM, as compared to NCI-H1688 and MRC-5 cells. At high concentrations of DN, the representatives of each subtype of NSCLC - A549, COR-L23 and NCI-H226 cell lines also showed the negative values of cell survival presented as LC_50_, thus indicating a net loss of cancer cells. Altogether, these data revealed that DN exerted selective antiproliferative activity (growth inhibition) and cytotoxic activity (cell death induction) against these NSCLC cancer cells.

In contrast to DN, paclitaxel showed a very strong antiproliferative activity with ~140-1270-fold higher GI_50_ and TGI values in all tested cell lines (except NCI-1688). However, paclitaxel appeared to exert selective growth inhibitory effects on NSCLC cancer cells but high cytotoxicity on human normal lung fibroblasts. Therefore, DN has potential as a promising anticancer agent because of its selective antiproliferative and cytotoxic activities without harming normal cells. Several studies described below were performed to provide insights into the molecular mechanism(s) underlying the anticancer activity of DN.

### The antiproliferative activity of DN in adenocarcinoma A549 cells

**Figure 2:**
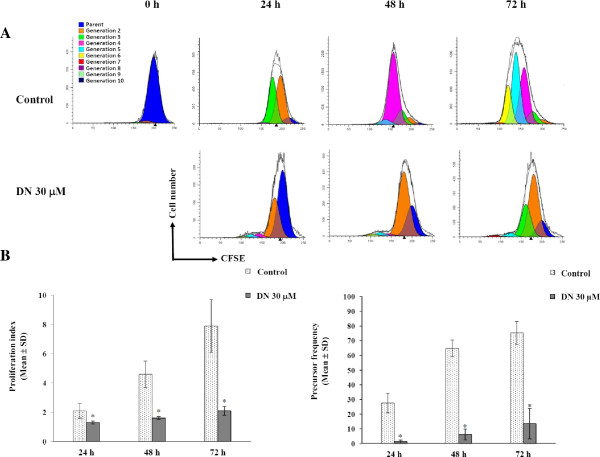
**Flow cytometric analysis of cell division by dilution of CFSE. (A)** CFSE histograms of control A549 cells (top) and DN-treated A549 cells (bottom) for 24, 48 and 72-h incubation period. A profile of sequential halving of CFSE fluorescence intensity with each generation was monitored, and numbers of cell division were analyzed using ModFit LT 3.2 program. **(B)** Proliferation index (left) and precursor frequency (right) of untreated and DN-treated A549 cells. Each bar graph represents the mean ± SD of at least three independent experiments. *Statistical significance (*p* <0.05) versus control.

As DN exhibited a growth inhibitory effect against all three subtypes of NSCLC with no statistical difference, we chose the adenocarcinoma cell line A549 to further investigate molecular mechanisms of DN’s antiproliferative and cytotoxic effects. The reason is that adenocarcinoma is the most common subtype, making up of 30-40% of all types of lung cancer. Unlike traditional methods of measuring cell proliferation, the CFSE cell division assay was employed to detect the cell division arrest induced by DN in A549 cells.As CFSE fluorescence intensity is reduced by one half at each cell division, the proliferation of CFSE-labeled cells either untreated or treated with DN can be monitored through such fluorescence intensity. Flow cytometric analysis of CFSE-labeled cells exposed to 30 μM DN using ModFit LT 3.2 program (Verity Software House, USA) revealed decreases in the number of generations of daughter cells with respect to untreated cells, indicating cell division arrest (Figure 2A). Evidently, the growth inhibitory effect of DM started from 24 to 48 h-long treatments. Moreover, the proliferation index of untreated cells reached approximately 2, 4 and 8 at 24, 48 and 72 h, respectively whereas the index values of DN-treated cells slightly increased in those time periods (Figure 2B). By 72 h, the proliferation index of these treated cells reached only 25% of that of untreated cells. Additionally, the untreated parental cells divided vigorously, resulting in substantial increases in precursor frequency that reached 75.3% after 72-h incubation. In contrast, only 13.6% of DN-treated parental cells underwent at least two rounds of cell division after 72 h, indicating that cell division greater than 85% of the A549 cells was hindered. Altogether, the results revealed DN-induced cell division arrest.

### The inhibitory effect of DN on cell cycle progression in A549 cells

**Figure 3:**
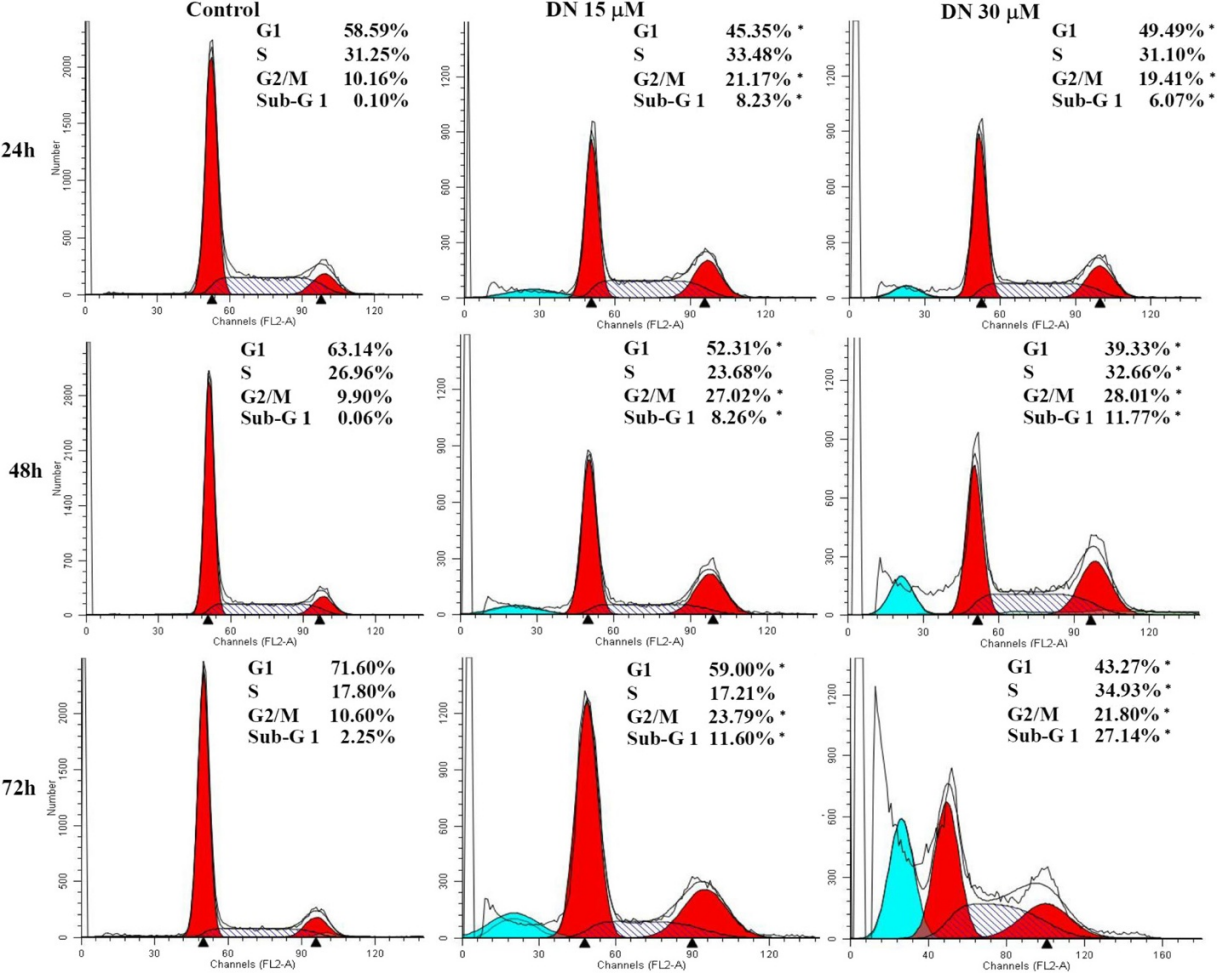
**Cell cycle distributions of DN-treated A549 cells by flow cytometric analysis.** A549 cells were either untreated or treated with 15 μM DN or 30 μM DN for 24, 48 and 72 h, then fixed, stained with PI, and analyzed by flow cytometry. Representative histograms from one of at least three independent experiments are shown. The percentages of cells in sub-G1, G0/G1, S and G2/M phases were analyzed using ModFit LT 3.2 program and indicated. *Statistical significance (*p* <0.05) versus control at equal incubation periods.

The effect of DN on cell cycle distribution was next assessed by flow cytometry. Significant alterations in the percentage of cells were observed in nearly all G1, S and G2/M phases of A549 cells treated with 15 μM and 30 μM DN (Figure 3) for 24, 48 and 72 h. In particular, the percentage of cells in the G2/M phase increased to the highest level in a 48-h treatment, thus indicating DN-induced G2/M-phase arrest. Such arrest was associated with a concomitant decrease in the percentage of cells in the G1 phase. Moreover, sub-G1 accumulation was observed in a dose- and time-dependent manner. The sub-G_1_ population was regarded as apoptotic cells containing only fractional DNA content. By 72 h, a marked increase in sub-G1 population in 30 μM DN treatment (~27% sub-G1) was accompanied by a decrease in the percentage of cells in the G2/M phase (Figure 3). These results indicated that the cell death was subsequent to the growth arrest. These results demonstrated that DN exerted its antiproliferative effect through cell cycle arrest at the G2/M phase and its cytotoxic effect through apoptotic death.

### Annexin V-FITC flow cytometric analysis of apoptotic effects of DN in A549 cells

**Figure 4:**
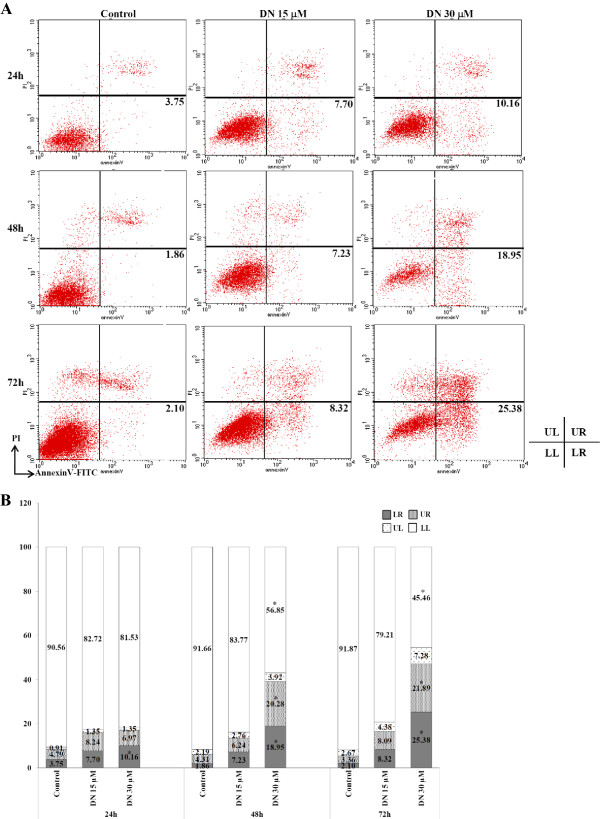
**Detection of apoptosis in DN-treated A549 cells by Annexin V-FITC flow cytometric analysis.** A549 cells were either untreated or treated with 15 μM DN or 30 μM DN for 24, 48, and 72 h, then stained with PI and AnnexinV-FITC, and measured by flow cytometry. **(A)** Representative dot plots from one of at least three independent experiments with the percentages of early apoptotic cells are shown. **(B)** Bar graphs with percentages of cells in the respective quadrants are indicated. LL: Viable cells, LR: Early apoptotic cells, UR: Late apoptotic cells, UL: Dead cells. *Statistical significance (*p* <0.05) versus control at equal incubation periods.

It was noted that cell cycle analysis first revealed the presence of apoptotic cells in DN-treated cells apart from G2/M-phase arrest. Thus, the next step in this study was to test whether apoptosis was indeed initiated in DN-treated cells. As translocation of phosphatidylserine (PS) to the external membrane leaflet is one of the earliest features of apoptosis, early apoptotic cells can thus be identified by the Annexin-V-FITC/PI double staining assay. Besides, double staining with PI allows differentiation of early apoptotic cells with intact membrane (AnnexinV ^+^/PI^-^) from late apoptotic/necrotic cells with leaky membranes (AnnexinV^+^/PI^+^) and normal cells (AnnexinV^-^/PI^-^). As a result, the different groups of these stained cells can be distinguished and quantified by flow cytometry.The Annexin-V-FITC/PI double staining assay indicated that DN induced a decrease in the percentage of viable cells (lower-left, LL) with a concomitant increase in percentage of early apoptotic cells (lower-right, LR) and late apoptotic cells (upper-right, UR) as compared to untreated cells (Figure 4A, B) in a dose- and time-dependent manner. However, the time-dependent effect of DN was significantly observed in 30 μM DN treatment, which significantly induced approximately 40% and 50% of early and late apoptotic cells combined at 48 h and 72 h, respectively (Figure 4A, B). These results confirmed that DN plays a critical role in triggering apoptotic cell death in DN-treated A549 cells. Notably, the increased early and late apoptotic cell populations were accompanied with the increased sub-G1 population in the cell cycle analysis after 48 and 72-h incubation of 30 μM DN.

### Effect of DN on caspase-3 activity in A549 cells

**Figure 5:**
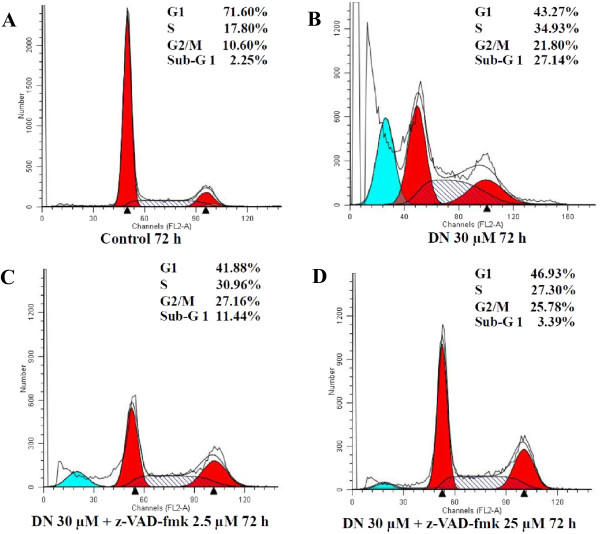
**Suppression of DN-induced apoptosis by the general caspase inhibitor z-VAD-fmk.** Representative histograms from one of at least three independent experiments depict cell cycle distributions. Distributions are shown in **(A)** for untreated A549 cells or in **(B)** for A549 cells treated with 30 μM DN or pretreated for 1 h with **(C)** 2.5 μM or **(D)** 25 μM pancaspase inhibitor z-VAD-fmk prior to the addition of 30 μM DN and further incubation for 72 h. The sub-G_1_ peaks (blue peaks) on DNA content histograms represent apoptotic cells and the percentages of cells in sub-G1, G1, S and G2/M phases are listed.

Caspases, a family of cysteine-aspartic proteases, are known to be crucial mediators in the apoptotic-signaling pathway [12]. To determine whether caspases are involved in DN-induced apoptosis, A549 cells were pretreated for 1 h with the pancaspase inhibitor z-VAD-fmk at 2.5 μM or 25 μM prior to the addition of 30 μM DN for 72 h and were measured for the subG1 population. The results demonstrated that z-VAD-fmk decreased the population of apoptotic cells according to its concentrations (Figure 5). Indeed, 25 μM z-VAD-fmk was sufficient to completely abolish DN-induced increase in the sub-G1 cells, thus indicating that caspases play a role in DN-induced apoptosis.

**Figure 6:**
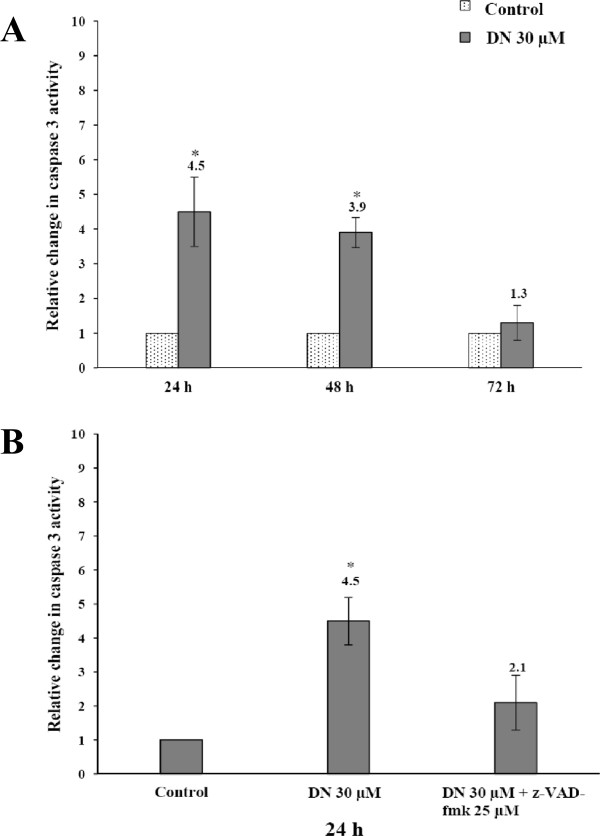
**Measurement of caspase-3 activity in DN-treated A549 cells. (A)** A549 cells were treated with 30 μM DN for 24, 48 and 72 h. **(B)** A549 cells were treated with 30 μM DN for 24 h or pretreated for 1 h with 25 μM z-VAD-fmk inhibitor prior to the addition of 30 μM DN and further incubation for 24 h. After these treatments, cell lysates were prepared, and enzymatic activities of caspase-3 were measured by the CaspACE™ colorimetric assay. Each bar graph represents the mean ± SD of at least three independent experiments. The caspase-3 activities in untreated cells are taken as 1-fold, and the figures on the chart display the relative changes of these activities in the treated cells. *Statistical significance (*p* <0.05) versus control at equal incubation periods.

As caspase-3 is well established as the major executioner caspase and its activation ultimately leads to cell death [13], it is thus suited as a read-out in an apoptosis assay. We next evaluated whether DN-induced apoptosis is associated with changes in caspase-3 activity. A549 cells treated with 30 μM for 24 h, 48 h and 72 h were analyzed for such activity using *in vitro* colorimetric substrate DEVD-pNA. Thirty μM DN was chosen because it induced prominent apoptotic effects. The results in Figure 6A showed a time-dependent activation of caspase-3. Its activity reached a maximum level within 24 h, then decreased over time, and was found to be not significantly different from the control at 72 h. On the contrary, the cell cycle results in the previous section revealed a maximum of ~27% apoptotic cells (sub-G1 peak) at 72 h (Figure 3). All these results indicated that the DN-induced caspase-3 activation started to increase many hours before a large population of apoptotic cells was observed. Pretreatment of A549 cells with 25 μM z-VAD-fmk prior to the addition of DN significantly decreased the caspase-3 activity to the same level as the control cells (Figure 6B). These results indicated that the DN-induced apoptosis was indeed triggered by the activation of caspase-3 in A549 cells.

### Effect of DN on mRNA expression of Bax and Bcl2 proteins in A549 cells

**Figure 7:**
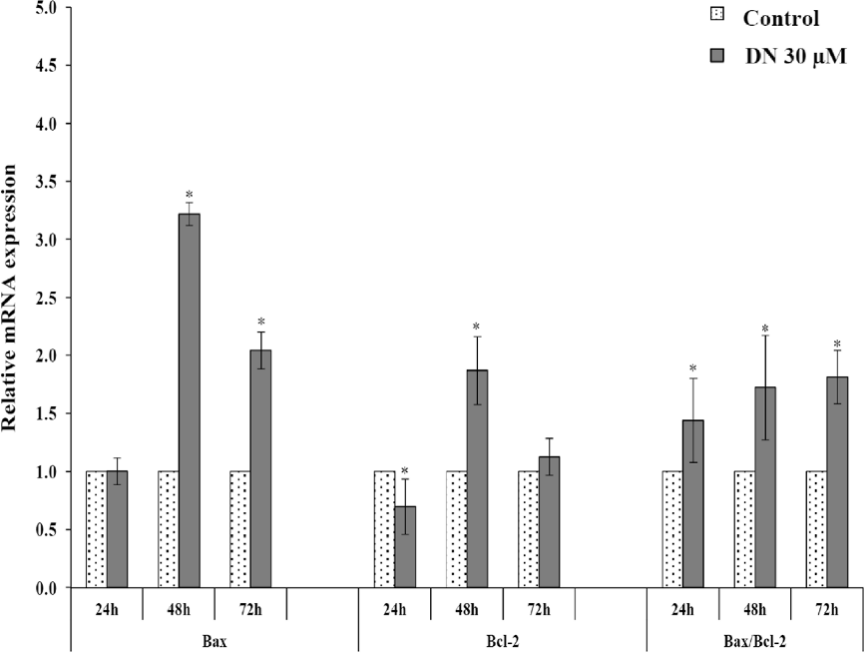
**Relative mRNA levels of Bax and Bcl-2 in DN-treated A549 cells.** A549 cells were treated with 30 μM DN for 24, 48 and 72 h. Total RNA was isolated and converted to single-stranded cDNAs, which were then quantified by real-time PCR using TaqMan probe and primer pairs pre-designed for Bax, Bcl-2 and GAPDH as an endogenous control. Relative mRNA levels of Bax, Bcl-2 and Bax/Bcl-2 ratios in treated A549 cells versus untreated cells are expressed as the mean ± SD of at least three independent experiments. *Statistical significance (*p* <0.05) versus control at equal incubation periods.

The Bcl-2 family proteins, which include pro-apoptotic Bax and anti-apoptotic Bcl2 proteins, have been reported to regulate mitochondrial membrane permeabilization (MMP), which subsequently triggers apoptotic pathways [14]. To determine whether such members are engaged in DN-induced apoptosis, the relative mRNA expression of Bax and Bcl2 was assessed by real-time quantitative RT-PCR. The results demonstrated that 30 μM DN caused Bax mRNA levels in treated A549 cells to increase over 3-fold and 2-fold at 48 h and 72 h, respectively (Figure 7). Similarly, Bcl2 mRNA expression increased nearly 2-fold after treatment of 30 μM DN for 48 h. However, it was noticeable that relative fold increases in the Bax mRNA level were higher than those in the Bcl2 mRNA level. At 72 h, the level of Bcl2 mRNA reduced to the same level as the control. Therefore, the increased Bax levels and the reduced Bcl2 levels caused high Bax/Bcl2 ratios (greater than 1) in a time-dependent manner, indicating DN-induced apoptosis via regulation of the Bcl-2 family protein-mediated mitochondrial pathway.

## Discussion

DN potentially exerted dose-dependent antiproliferative (described as GI_50_ and TGI values) and cytotoxic effects (represented as LC_50_ values) only on the three subtypes of NSCLC cell lines, but not on the SCLC cell line. The differential sensitivity of these cancer cells to DN could be explained in part because of their distinctive molecular characteristics [3]. Studies on molecular mechanisms of DN in regard to its antiproliferative effect revealed significantly low proliferation index and precursor frequency values in DN-treated A549 cells via G2/M-phase cell cycle arrest. Several anticancer agents have been noted to mediate such G2/M phase arrest in cancer cells through multiple mechanisms: downregulation of CDK1-cyclin A/B complexes, inactivation of Cdc25C activity, and disruption of tubulin polymerization and spindle assembly [15, 16]. In this regard, the effects of DN on the types and levels of these key regulators need to be identified.

In addition to the antiproliferative effect, significant net loss of cell viability in NSCLC cells at high concentrations of DN in the SRB assay indicated its cytotoxic effect. Such data is in accordance with the results obtained by flow cytometric analysis, which determined the presence of apoptotic cells reflected by the sub-G1 DNA peak and early (AnnexinV ^+^/PI^-^) and late (AnnexinV^+^/PI^+^) apoptotic cells in DN-treated A549 cells. In this context, Annexin V is a recombinant PS-binding protein that interacts strongly and specifically with PS residues and is typically used in conjunction with PI to distinguish early from late apoptotic cells [17]. All these findings demonstrated apoptosis-involved mechanisms underlying cytotoxicity of DN for the first time. Although both late apoptotic and necrotic cells are Annexin V and PI-positive, the presence of these cells with early apoptotic cells suggests that such dead cells resulted from an apoptotic process rather than a necrotic process.

The apoptotic caspases appear to be activated in a protease cascade in which the activated apical caspases responding to apoptotic stimuli directly activate the executioner (effector) caspases in a precisely controlled process [18]. Among these effector caspases, caspase-3 plays a central role in the execution phase of both the intrinsic (the mitochondrial) and extrinsic (the death receptor) pathways of apoptosis by cleaving many key cellular proteins, such as poly (ADP ribose) polymerase (PARP), inhibitor of caspase-activated DNase (ICAD), and various other proteins [19]. This cleavage mediates disassembly of the cell into the typical apoptotic morphological changes, such as cell shrinkage, chromatin condensation, membrane blebbing, and DNA fragmentation [5]. In this study, the pancaspase inhibitor z-VAD-fmk, which irreversibly binds to and blocks the cleavage site of the caspases, was able to completely abrogate the DN-induced increase in the sub-G1 cell population and DN-induced activation of caspase-3 in A549 cancer cells. These results strongly indicated that DN-induced apoptosis was executed mainly through a caspase-dependent pathway. In addition to such a pathway, the execution of apoptosis also constitutes a caspase-independent pathway that is mediated by mitochondrial proteins AIF [20] and EndoG [21].

Pro-apoptotic (e.g. Bax) or anti-apoptotic (e.g. Bcl-2) proteins of the Bcl-2 family, a group of structurally related proteins, play a critical role in regulating the permeability of the outer mitochondrial membrane (OMM) [22]. Indeed, an increase in the ratio of Bax/Bcl-2 causes the permeabilization of OMM, leading to a hierarchical release of the apoptogenic proteins such as cytochrome c, Smac/Diablo and Omi/HtrA2, and subsequently resulting in downstream caspase-3 activation [22, 23]. Increases of Bax/Bcl-2 ratio in this study suggest that the susceptibility of DN-treated A549 cells to apoptosis could be modulated, at least in part, through the mitochondrial pathway.

Besides new insights on the anticancer mechanisms of DN, understanding its bioavailability when consumed per ounce (oz) also helps determine the amount of DN to be taken orally in compliance with the DN concentrations examined in this study. However, no such data are available, and *in vivo* oral bioavailability of DN needs to be further evaluated.

## Conclusions

In this study, DN selectively inhibited the growth of A549, COR-L23 and NCI-H226 representative cell lines for each of three subtypes of NSCLC. In adenocarcinoma A549 cells, DN induced G2/M-phase cell cycle arrest and apoptosis, at least in part, via mitochondrial membrane permeabilization mediated by Bax and Bcl-2 proteins, leading to caspase-3 activation. The present study greatly contributes to the understanding of the anticancer activity of DN for the first time and also provides evidence that DN deserves additional evaluation as a natural anticancer agent for human NSCLC.
